# Selected Aspects of Electrochemical Micromachining Technology Development

**DOI:** 10.3390/ma14092248

**Published:** 2021-04-27

**Authors:** Sebastian Skoczypiec, Piotr Lipiec, Wojciech Bizoń, Dominik Wyszyński

**Affiliations:** Chair of Production Engineering, Cracow University of Technology, al. Jana Pawla II 37, 31-864 Krakow, Poland; piotr.lipiec@pk.edu.pl (P.L.); wojciech.bizon@pk.edu.pl (W.B.); dominik.wyszynski@pk.edu.pl (D.W.)

**Keywords:** electrochemical machining (ECM), electrochemical micromachining (ECMM), pulse electrochemical micromachining (PECMM), localization factor, universal tool, technology

## Abstract

The paper focuses on the fundamentals of electrochemical machining technology de-elopement with special attention to applications for micromachining. In this method, a material is removed during an anodic electrochemical dissolution. The method has a number of features which make it attractive technology for shaping parts with geometrical features in range of micrometres. The paper is divided into two parts. The first one covers discussion on: general characteristics of electrochemical machining, phenomena in the gap, problems resulting from scaling down the process and electrochemical micromachining processes and variants. The second part consists of synthetic overview of the authors’ research on localization of pulse electrochemical micromachining process and case studies connected with application of this method with use of universal cylindrical electrode-tool for shaping cavities in 1.4301 stainless steel. The latter application was conducted in two following variants: electrochemical contour milling and shaping carried out with sidewall surface of rotating tool. In both cases, the obtained shape is a function of electrode tool trajectory. Selection of adequate machining strategy allows to obtain desired shape and quality.

## 1. Introduction

In electrochemical machining the material is removed during an anodic electrochemical dissolution which occurs due to electrochemical reactions taking place during the electric current flow through thin interelectrode gap filled with electrolytic solution. The history of ECM technology starts in the beginning of the 20th century; however, these machining methods are still classified as non-conventional or non-traditional. The process, developed in 1928 by Gusev and Rozhkov [1], is based on the assumptions that (1) the electrode-tool (cathode) is moved towards the workpiece (anode) with the same feed rate as dissolution process ratio, and (2) the electrolyte flow is forced between the electrodes. It allows to carry out dissolution in the thin interelectrode gap (<1 mm) and maintain relatively high electric current density (>10 A/cm^2^). Further developments were focused on proper selection of machining parameters and lead to modern electrochemical machining technology [2,3,4]. Considering dimensions of a machined area, one can propose the following classification:

- electrochemical macromachining (shaping area > 100 mm^2^),
- electrochemical mezomachining (shaping area in range 1–100 mm^2^),
- electrochemical micromachining (shaping area < 1 mm^2^).

Such classification results not only from the machined area dimensions but is also related to specificity of technology design and technical requirements for machine tools and process control. The paper focuses one on the third area of application listed above, which stands out among other micromachining methods due to: (1) the possibility to remove material with negligible mechanical and thermal interaction (no workpiece and tool deformation) and (2) machining resolution in range of nanometers.

The recent research trends in field of electrochemical micromachining are focused on the increase of resolution and precision of machining. It is related to selection of optimal conditions of dissolution under the small interelectrode gap (<100 μm). The scope of the conducted research includes especially: (1) explanation of the phenomena in interelectrode gap during dissolution [5]; (2) analysis and modification of electric current distribution on the workpiece surface [6,7]; (3) selection of optimal composition of electrolyte [8]; (4) technology development, i.e., simulation of machining or the tool shape/path design [9]; (5) development of power suppliers and gap control strategy [10]; and (6) improvement of machining conditions by additional energy sources (i.e., ultrasonic vibrations [11,12]) or (7) integration with other technologies [13].

One can state that electrochemical macro-, mezzo-, and micromachining processes are based on the same physical principles. However, due to the size of machined area and differences connected with gap thickness, some unique effects related to scaling-down the electrochemical system are observed. In micromachining the dissolution localization is of particular importance and there is no universal methodology to evaluate it empirically. Definition and empirical determination of dissolution localization factor may allow to indicate limits of accuracy for given machining parameters.

The objective of the analysis and research presented in this paper is to outline the problems connected with electrochemical micromachining technology. The paper was divided into two following parts:

- Literature-based discussion focused on physicochemical and technological fundamentals of electrochemical micromachining technology development. This part covers: general characteristic of ECM process, phenomena occurring in the interelectrode gap during shaping, problems of ECM adaptation to micromachining, with discussion on necessary conditions for downscaling of the process and the machining system, and description of ECMM processes and variants.
- Experimental part which consists of synthetic overview of the authors’ research on localization of pulse electrochemical micromachining process and case studies on application of pulse electrochemical micromachining with universal cylindrical electrode-tool.

The described in the paper research were conducted in order to translate results of the basics research on electrochemistry to technical language. It enabled understanding the impact of the physical and electrochemical phenomena on the limitations of manufacturing process. This is a step toward to fill the gap between basic and applied research to gain synergetic effect.

## 2. General Characteristic of Electrochemical Machining

In ECM, mechanism of material removal is based on Faraday’s law of electrolysis. The difference in electric potential between electrodes (voltage *U*(*t*)) makes electric current flow through the interelectrode gap. Due to electrochemical reactions that occur on the electrodes–electrolyte interfaces, the outer electronic conduction is changed to ionic. One of these reactions is dissolution of a workpiece (anode) material. Based on Faraday’s law, the efficiency of ECM depends on amount of charge that flows through the gap and chemical composition of workpiece material. Therefore, the process is unrelated to mechanical properties of the machined material. Some other, attractive from micromachining application point of view, advantages of this process may include [4,14]: (1) negligibly small mechanical and thermal load during the process, (2) lack of tool wear and (3) negligible changes in the structure, micro hardness, and chemical composition in the surface layer of the workpiece.

The exact course of electrochemical phenomena depends on voltage characteristic, materials of electrodes (workpiece-anode, tool-cathode), and composition and physical parameters of the electrolyte. Dissolved ions of the workpiece material take part in further chemical reactions. Moreover, some gas generation and bubble evolution occur on the surface of the electrode-tool, i.e., for water-based electrolyte, hydrogen accumulates near tool surface. These products of electrochemical reactions change physical properties of the electrolyte, which has negative impact on a shaping process. Additionally, during machining, Joule heat, proportional to electric current intensity and the interelectrode gap resistance, appears and is dissipated in the electrolyte. Therefore, in the machining gap the distribution of physical properties of the electrolyte is heterogeneous. The temperature, pressure, flow velocity, pH and conductivity are changing randomly. It affects the output machining parameters such as gap thickness *S* and surface geometric structure (i.e., Ra, Rz, and Rq). They can be considered as random variables described by averages and standard deviations, and their values can be estimated only with specified probability less than 1. 

In the case of machining with constant voltage and tool feed rate (*U*(*t*) = *const* and *v_f_*(*t*) = *const*), the range of conditions when machining is stable and uniform over workpiece area is very narrow. When machining is conducted in conditions optimal for accuracy and surface quality, the gap thickness should be small (*S* < 0.1 mm). In such cases, to avoid critical states in the gap, dissolution conditions should be regularly restored; to this end, certain discrete ECM variants were developed. Breaks necessary to restore gap conditions during the machining can be implemented by a tool vibration (oscillation), pulsed voltage or by some combinations of these solutions concurrently [4,15]. In pulse electrochemical machining (PECM), dissolution takes place during voltage pulse *t_i_*, and dissolution products are removed from the gap during the pause *t_p_*. The applied pulse times are in a range from 10^−2^ to 10^−9^ s and depend on PECM technological variant (see paragraph 5). Furthermore, to improve electrolyte exchange, a variety of kinematic solutions (i.e., vibrations or periodical gap re-adjustment) are used. Discrete variants of electrochemical machining can improve machining accuracy and surface layer quality by decreasing heterogeneity of physical conditions along machining gap and decreasing the gap size where machining is stable. Diminishing of gap size increases current density and allows to use the relation between electrochemical machinability and current density to localize dissolution process [16].

Recent developments in ECM, especially in the area of PECM, allows to apply this method to accurate shaping of 3D structures with machining area from several to tens square millimetres with accuracy similar to milling or EDM sinking [17,18,19,20,21,22]. Due to development and improvements of machine-tools, power suppliers, electrolyte stations, and control systems, the technological borders of PECM were shifted toward more accurate and precise machining. The decrease of gap thickness significantly below 100 μm allows for shaping of more accurate 3D structures with relatively high efficiency (i.e., sinking process takes place with feed rate 0.1–3 mm/min). Therefore, in recent years the application of ECM has been extended to new areas of precise machining: precise mechanics, jewellery, optical instruments, chemical devices, and the medical and pharmaceutical industry. Everywhere the method of machining of small, sculptured, precise and made of difficult-to-cut materials parts is needed. Above mentioned advantages and developments of ECM technology made application of ECM profitable also for mid-series or even in some cases for small-series production.

## 3. Phenomena in the Interelectrode Gap during the Electrochemical Shaping

**Figure 1:**
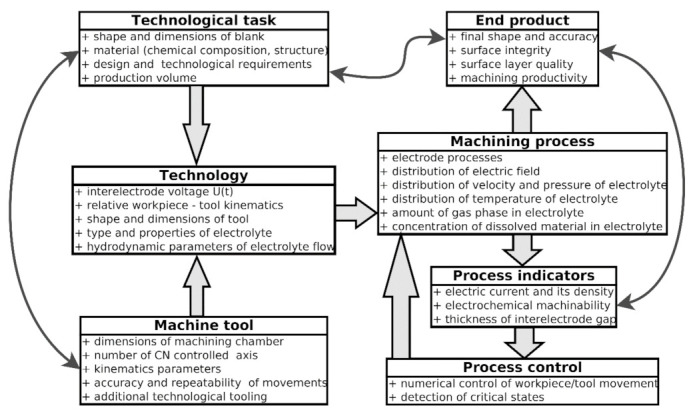
Scheme of the essential interrelationships of electrochemical machining process [23].

The process of electrochemical dissolution is conducted in the interelectrode gap. This is a part of the complex system consisting of a machine tool, clamping gauge, workpiece, and tool. The principle of this process is based on electrophysical phenomena and, in aspect of technological task, interrelationships presented in Figure 1 are essential. The goal of the machining (or technological task) is to produce a series of parts made of specified material which meet requirements of the design. The production should be cost-effective; therefore, it should be executed with desired productivity and energy consumption. Depending on these requirements, the choice of appropriate ECM technology means choosing optimal type of the interelectrode voltage (direct or pulse), relative workpiece-tool movement, selection of electrolyte (type and concentration), and electrode-tool shape, and dimensions. One can state that such decisions strictly depend on the specification of a machine-tool. This is because dimensions of a machining chamber (or table) and possibilities of additional technological tooling installation limit possibilities of technological task implementation.

The conditions of electrochemical shaping are affected by:

- type and properties of the electrolyte (electric conductivity and its relation to the changes of temperature, specific heat, density, and dynamic viscosity);
- chemical composition and structure of machined material;
- machining parameters (electrode feed rate, interelectrode voltage, and pressure of the electrolyte at the gap inlet and outlet);
- electrochemical characteristic of the tool-electrolyte-workpiece system (described by relations of electrochemical machinability and electrodes’ potential drop dependent on current density and electrolyte flow velocity);
- geometrical relations between tool and workpiece, i.e., type of electrode tool (shape, dimensions, design details, and way of electrolyte supply to machining area), initial shape of the machined surface, initial position of the electrode tool and workpiece, parameters of relative tool-workpiece movement.

The above mentioned factors can be divided in three categories, i.e., fixed, controlled, and disturbing. They have influence on machining phenomena, its course and the final shape and properties of workpiece.

The fixed factors include workpiece and electrode tool properties, shape and dimensions of electrode tool and type of the electrolyte. The controlled factors have the strongest impact on the process of dissolution and the possibility of gap size regulation. This group includes: the electrode tool feed rate, the interelectrode voltage (direct or pulse), the voltage pulse characteristic and the gap size. It is characteristic for ECM process that majority of controlled factors are interdependent.

The course of ECM is also influenced by a number of disturbing factors. The most important are instability of the electrolyte flow, heterogenous composition of the machined material, structural and chemical properties, instability of power supply, and the machine tool vibrations.

The result of electrochemical shaping depends on the above mentioned factors and relations among them. During the ECM, the following complex physical and chemical phenomena take place in the machining gap: anodic dissolution, changes of the electric field distribution, changes of potential on the electrode–electrolyte interface, changes of the electrolyte pressure and velocity distribution, changes of the electrolyte chemical and electric properties, heat dissipation, and chemical reactions. Electrochemical dissolution is accompanied by dissipation of significant amount of heat. Therefore, reliable forced electrolyte flow plays crucial role during the process (electrolyte also removes dissolution products). It is noteworthy that appropriate hydrodynamic conditions in the interelectrode gap prevent unfavorable machining phenomena which define critical states in this volume. Calculation of pressure and velocity field in the gap is difficult (in many cases also impossible) because the electrolyte flow through the gap is three-dimensional, turbulent, multiphase, and random. Irregular distributions of the temperature, gas concentration, and electric conductivity of electrolyte have impact on the process factors such as gap size, electric current intensity and its density and electrochemical machinability. Values of these factors (also accuracy of prediction) determine the results of the machining which could be described with technological indicators of surface integrity, accuracy of machining, and material removal rate.

As a result of the above-mentioned phenomena, a final part described with certain surface integrity (structure, physical, and chemical properties of the surface layer) and geometrical accuracy is produced.

## 4. ECM Process Adaptation to Micromachining: Localization of Anodic Dissolution

**Figure 2:**
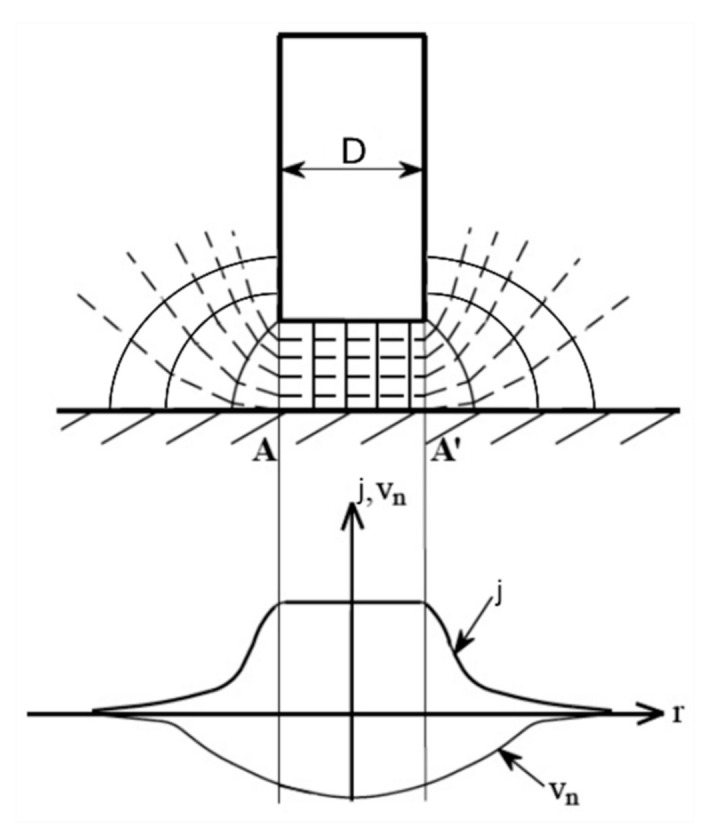
Scheme of machining area showing the electric field intensity distribution, corresponding current density *j* and electrochemical dissolution ratio (speed *v_n_*) distribution; *D*—tool diameter.

One of the conditions for successful adaptation of machining process to micromachining is decreasing of unit removal (UR). It is defined as a part of the workpiece removed during one cycle of removal action [24]. UR determines the limit of the smallest adjustable dimensions of the part, so UR of sub-micrometre range is required when the object size is very small or when high precision of the product is required. In ECM, material is removed by electrochemical dissolution and theoretically the smallest UR is a single ion. However, achieving high localization of the dissolution process remains a key problem. It results from distribution of electric field in the machining area (see scheme in Figure 2) and is called as delocalization effect.

**Figure 3:**
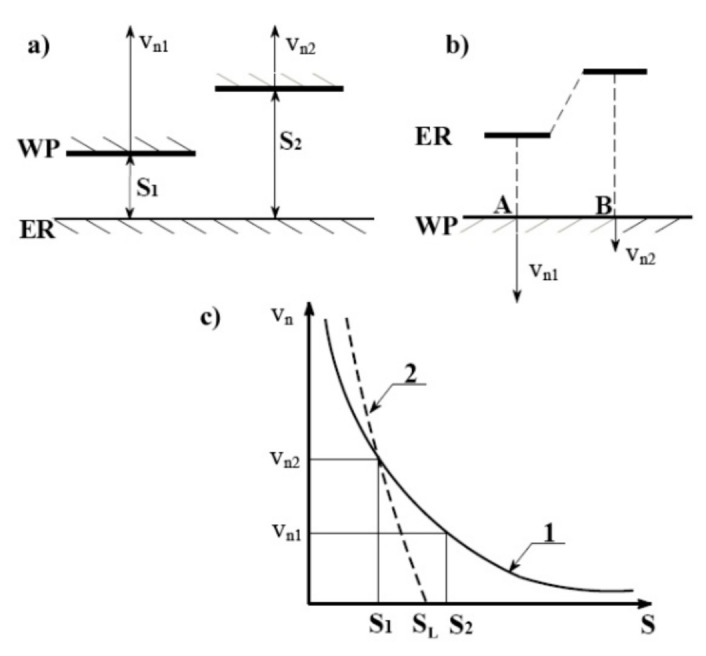
Scheme of the ECM localization idea for flat tip electrode (**a**) and shaped electrode (**b**) (WP—workpiece surface, ER—electrode surface); (**c**) relation of dissolution velocity *v_n_*(*S*) to interelectrode gap size *S* (*S_L_*—limiting interelectrode gap thickness) [17].

During the process, the distance between the electrode tool and the workpiece is equal to interelectrode gap size *S*, but the area of anodic dissolution spreads over much greater distances than gap size *S* and tool dimeter *D*. This results in the area of dissolution being larger than the area of the electrode tool tip (Figure 2) and limits possibilities of scaling down the ECM process (as some problems with pitting and surface quality can occur due to the change of electrochemical dissolution conditions in a transitional area.). Distribution of the electric field intensity determines primary current density distribution and results from the electrode and workpiece geometry. However, it does not provide satisfactory localization. Therefore, to obtain high localization of the process, the current density distribution has to be modified [4]. According to [25,26], the measure of this modification is defined by the degree of anodic dissolution localization. It can be defined based on relation *v_n_*(*S*) (Figure 3). In case of ideal (non-modified, curve 1 in Figure 3c) dissolution process, one can state that:

(1) $$v_{n}(S)=\frac{const}{S^{n}}$$

therefore, for *S*_2_ = 10*S*_1_ dissolution speed *v_n_*_2_ has a relatively high value (10% of *v_n_*_1_).

In the Figure 3c, curve 2 describes the process of high dissolution localization in which for *S* > *S_L_* dissolution process does not occur. So, the relation between $\frac{v_{n1}}{v_{n2}}$ and $\frac{S_{2}}{S_{1}}$ for highly localized dissolution process is as follows [17]:

(2) $$\frac{v_{n1}}{v_{n2}}={\left(\frac{S_{2}}{S_{1}}\right)}^{n}$$

and in such a case factor *n* can be considered as the measure of the localization:

(3) $$n=log_{\frac{S_{2}}{S_{1}}}\left(\frac{v_{n1}}{v_{n2}}\right)$$

For model situation (curve 1 in Figure 3c) *n* = 1, in order to localize the process, *n* value should be as high as possible. The slope of the curve *v_n_*(*S*) should be as steep as possible and in such a case, dissolution takes place in limited area and, for *S > S_L_*, it does not occur.

**Figure 4:**
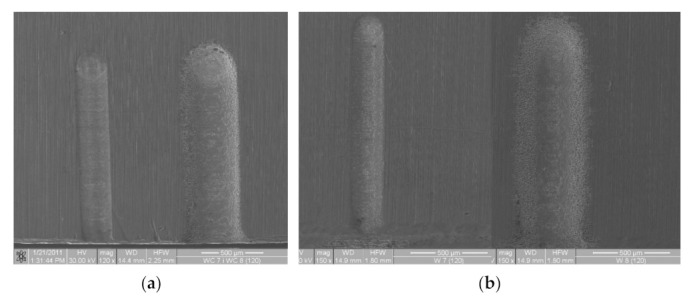
Comparison of the surfaces of the grooves obtained as a result of a single pass of electrode tool; workpiece material: 1.4301 stainless steel, electrode material: (**a**)—WC, (**b**)—W; electrode diameter *D* = 0.2 mm; machining parameters: velocity *v_p_* = 375 μm/s, *t_on_* = 600 ns, interelectrode voltage *U* = 6 V (left) and *U* = 24 V (right) [23].

The most effective approach to improve *n* number value is to apply pulse voltage (pulse electrochemical machining, PECM) [17]. However, it is notable, that when machining parameters are improperly selected, even for PECM significant delocalization effect can occur. Some examples were presented in Figure 4. The localization of PECM can also be evaluated based on the volume of the material removed during one pulse which, according to the relation $V_{p}=\eta k_{v}Q\left(t\right)$, is proportional to electrical charge *Q*(*t*) transported through the gap during pulse time *t_i_*. Therefore, according to [27] the main characteristic of PECM can be expressed as:

(4) $$q\left(t\right)=\frac{A}{S^{n}}$$

where *q*(*t*) is charge density and *n* is localization factor which characterizes PECM process. To achieve good, localized dissolution, *n* value should be as high as possible.

Our research on the localization factors defined by Equations (3) and (4) were presented and discussed in paragraph 6.

## 5. Processes and Variants of Electrochemical Micromachining

**Figure 5:**
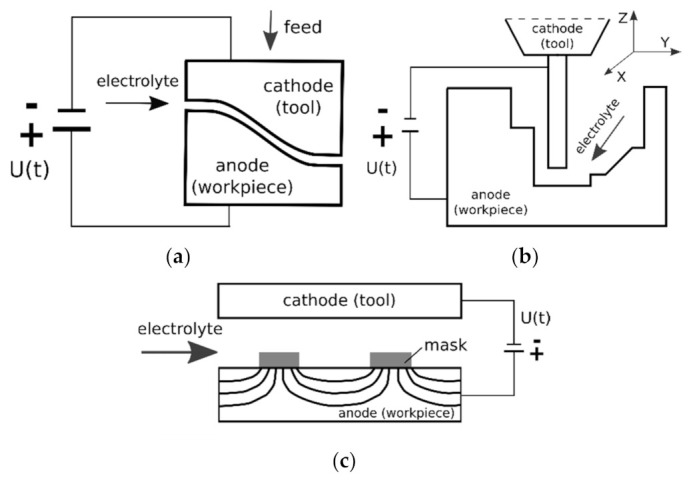
Variants of electrochemical micromachining: (**a**) sinking, (**b**) machining with universal electrode tool, and (**c**) through mask electrochemical micromachining [28].

The group of electrochemical micromachining processes includes cathodic (i.e., electrodeposition, electroplating, electroforming, through-mask electroplating), anodic (i.e., electrochemical micromachining, electroetching, and electropolishing) or open circuit processes (chemical polishing, chemical milling, and chemical etching) [28]. Majority of these methods were developed to shape planar structures and only electrochemical micromachining (ECMM) is suitable for manufacturing 3D complex shapes. In ECMM, the allowance can be removed in following variants:

- sinking—which is based on tool shape reproduction in the machined material (Figure 5a);
- machining with universal electrode tool (Figure 5b);
- through-mask shaping (Figure 5c).

**Figure 6:**
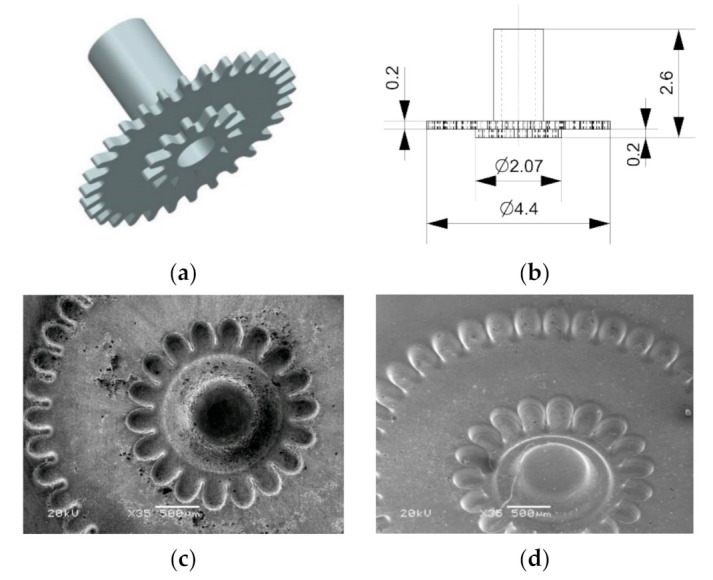
Example of electrochemical sinking application: (**a**) electrode tool 3D model; (**b**) scheme of the electrode tool; inner structure machined in 1.4301: (**c**) steel; (**d**) pure nickel [32].

During the electrochemical sinking, the electrode tool moves towards the workpiece and the obtained shape depends on the dimensions of the electrode tool and size of the interelectrode gap. The process development is connected with solving the problem of effective gap flushing and costly production of electrode tool with sophisticated shape. Therefore, the range of application of sinking operation in micromachining is limited to manufacturing cavities or holes with simple cross-section (i.e., cylinder, and polygon) and is preferred in mezzo-manufacturing (i.e., where machined area is in range of mm^2^) (example in Figure 6). Through-mask electrochemical micromachining was developed especially, to generate the micro-dimples array with controlled shape and density and this process also includes masking to protect selected areas of workpiece from dissolution [12,29]. Therefore, in micromachining application dominant are the operations where simple electrode-tool (i.e., cylindrical with spherical, or flat or tip, wire) can be applied [30,31]. The process is similar to milling and the machined shape results from the electrode-tool path. Thus, dimensions and the final shape of the part depends on the diameter of the electrode-tool, the size of the interelectrode gap, and repeatability and accuracy of the tool movement. The shaping in electrochemical milling regime can be conducted with frontal or lateral surface of the electrode-tool. This variant is commonly used in micro-tooling industry (shaping electrodes, dies, blanking dies, stamps, etc.). However, a complicated control system of electrode-tool path and interelectrode gap can be mentioned as a disadvantage of electrochemical milling.

As it was mentioned before, the most efficient solution to scale down ECM process is to apply the pulses of voltage. Depending on pulse range, the following reasons for applying pulse voltage could be defined: (1) to avoid the critical conditions in the gap (*t_i_* > 1 ms); (2) to use relations *ηk_v_*(*j*) and *j*(*S*) to localize anodic dissolution in areas where the gap is smaller (*t_i_* < 1 ms, *ηk_v_*—electrochemical machinability, *S*—interelectrode gap size); and (3) to use transient phenomena (time of the electric Helmholtz double layer charging to the activation overpotential) to define spatial machining resolution (in such cases pulse time *t_i_* < 500 ns—determines how far from the electrode tool the workpiece surface is activated). The last variant, the nanosecond pulse, allows to obtain the best machining resolution. However, flexibility of this method is limited because the results of machining depend on electrode tool size, workpiece material composition, and heterogeneity of the structure. It is also crucial to precisely select the electrolyte composition and its additives for each machining material [8]. Therefore, in spite of high potential of dissolution localization, nanosecond pulse machining is not widely used in the industry.

## 6. Research on Localization of PECM Process

### 6.1. Materials and Methods

**Figure 7:**
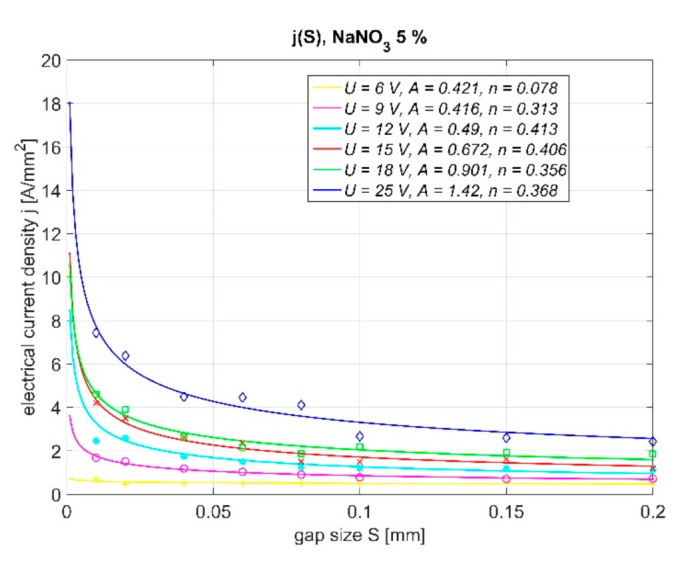
Set of *j*(*S*) characteristic for electrolyte NaNO_3_ 5%.

**Figure 8:**
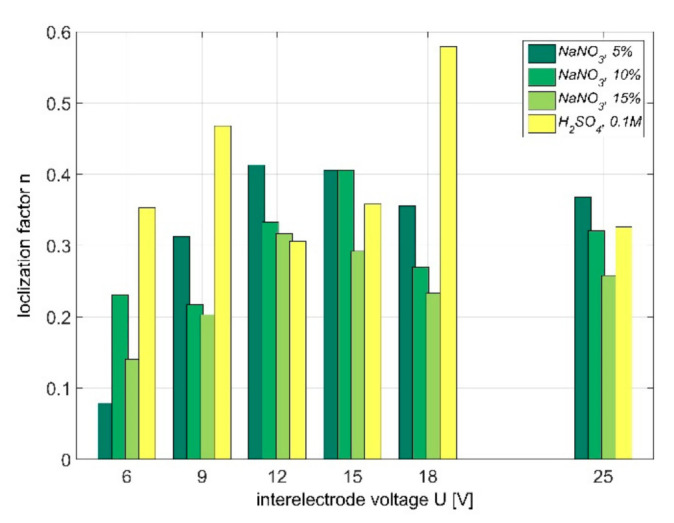
Localization factor *n* for NaNO_3_ and 0.1 M H_2_SO_4_ [27].

Theoretical considerations presented in paragraph 4 can be the basis for experimental research. In order to determine localization of PECM process, the material removal during one pulse should be determined. The methodology of determining the localization factor during PECM was presented in [27]. The analysis presented there was limited to linking the calculated with Equation (4) value of *n* with real conditions of machining. The calculations were based on empirical relations *j*(*S*) (Figure 7) and the obtained values of *n* were summarized in the Figure 8. Thus, for the investigated set of parameters one can state that:

- the localization of the process depends on pulse voltage *U* (the lowest value of *n* was obtained for *U* = 6 V and *U* = 9 V and the highest *n* values were for *U* = 12 V, 15 V, and 18 V; however, the results depend on the applied type of electrolyte);
- the localization is also connected to hydrodynamic phenomena in the gap. For PECM, pulse turn on is followed by electrolyte pressure increase in the gap, which supports evacuation of dissolution products (this effect intensifies with voltage increase).

However, it is worth to underline that during this experiment some problems related with efficient removal of the products of electrochemical reactions were observed. It implies significant inaccuracy in approximation of empirical relation *j*(*S*) with theoretically obtained Equation (4). The observed electric current increase with gap size decrease is not as steep as predicted by model. The discrepancies concern mainly the values obtained for small gaps sizes (*S* < 0.05 mm) and are connected with problems with effective gap flushing during the experiment. Therefore, interpretation of the results presented in Figure 8 is challenging.

In this paper, the results of the experiment presented in [27] were analyzed with regard to the characteristic *q*(*S*,*U*) which describes charge density in function of gap size *S* and interelectrode voltage U. According to Equation (3), such a relation allows to connect technological parameters with localization of dissolution process. The research was performed for microsecond PECM (*t_i_* = 1 μs, *t_p_* = 10 μs), six levels of voltage amplitude (6 V, 9 V, 12 V, 15 V, 18 V, and 25 V), seven levels of gap size (0.01 mm, 0.02 mm, 0.04 mm, 0.06 mm, 0.08 mm, 0.1 mm, 0.15 mm, and 0.2 mm) and two types of electrolytes: water solution of NaNO_3_ (5%, 10%, and 15%) and H_2_SO_4_ (0.1 M). The results were analyzed taking into account the response surface *q*(*S*,*U*) assumed as exponential function of gap *S* and voltage *U*:

(5) $$q\left(S,U\right)=e^{f\left(S,U\right)}$$

where function *f*(*S*,*U*) is a square polynomial. The regression function was determined with application of statistic toolbox of Matlab ver. R2014b software (MathWorks, Natick, MA, USA).

### 6.2. Results

**Figure 9:**
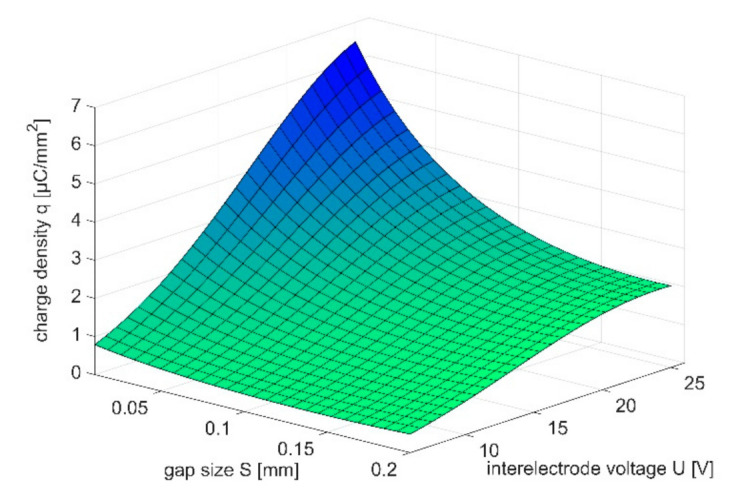
Relation *q*(*S*,*U*) for 5% water solution of NaNO_3_ as electrolyte.

**Figure 10:**
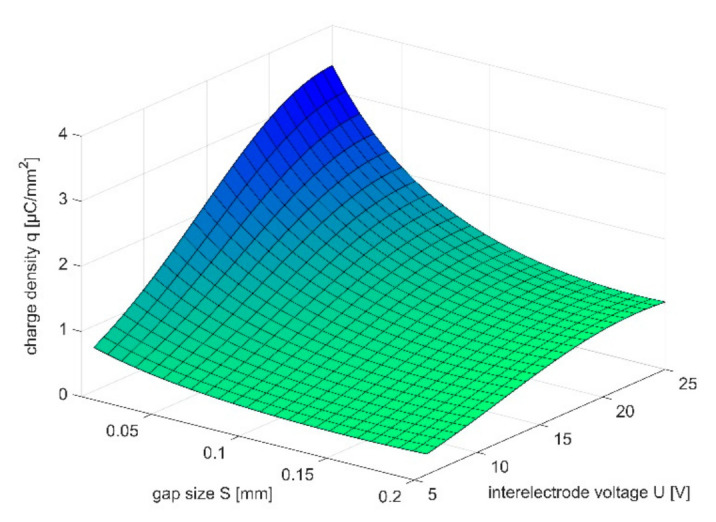
Relation *q*(*S*,*U*) for 0.1 M water solution of H_2_SO_4_ as electrolyte.

The examples of obtained response surfaces were presented in Figure 9 and Figure 10. Based on obtained characteristics, one can state, that: (1) the charge density *q* increases with square of gap size *S*; (2) the influence of inter-electrode voltage *U* on charge density *q* is significant especially when *S* < 0.1 mm (it is connected with change of electrolyte conductivity *κ* due to temperature increase). 

The exponential relation *q*(*S*,*U = const*) allows to estimate impact of interelectrode voltage *U* and gap size *S* on localization factor *n*. Taking into account the relation

(6) $$v_{n}\left(S\right)=\frac{\eta k_{v}q\left(S\right)}{\left(t_{i}+t_{p}\right)},$$

one can calculate localization factor according to relation (3) for selected range of gap size *S*_1_–*S*_2_. Example of such calculations were presented in Table 1 and Table 2.

The obtained results proved that, in case of machining in current limited state, localization of the dissolution increases with *U* (*v_n_*_1_/*v_n_*_2_ and *n* increases with *U*). Localization factor is also connected with range of gap sizes *S*_1_–*S*_2_. Comparing the results of the machining in these two ranges of *S*_1_–*S*_2_: 0.01–0.1 mm and 0.01–0.2 mm, one can state that in the second case the calculated *v_n_*(*S*) curve slope *dv*/*dS* is significantly higher. Thus, assuming that the gap size, resulting from the process parameters, lies in one of the above-mentioned ranges, better machining precision should be expected in the second case. This is because in this case smaller differences in machining speed for *S_min_* and *S_max_* are observed. Generalizing, the technologist should focus to execute the machining process in the narrowest possible range *S_min_*–*S_max_*, while minimizing the value of *S_min_*. This conclusion is crucial for the electrode-tool design, because in the described conditions, its shape may be close to final part equidistant. However, when it is impossible to provide uniform gap size distribution along the whole machined area and the difference between *S_min_* and *S_max_* is high, a better solution is to apply smaller value of voltage *U*, so the dissolution speed (and electrode tool feed) in the gap is not very high.

Basically, decrease of pulse time allows to conduct machining with smaller size gap, which also means that electric current density and *v_n_* increase too. The dynamics of the increase of *dv_n_*/*dS* depends on the voltage pulse time (the shorter pulse—better localization), what, together with increase of U, can cause problems with excessive electrolyte temperature increase in the gap. Therefore, fulfilment of the above-mentioned conditions of good localization (i.e., small gap, high voltage, and short pulse time) constitutes a kind of multi-criteria optimization with target function as compromise between precision and efficient machining.

It has to be emphasized that, defined by the Equation (3), localization coefficient *n* cannot be used as a measure to compare machining cases with different ranges of gap size. Such a comparison is possible based on localization coefficient defined by the Equation (4). The type of approximation function *q*(*S*), proposed in such a way, allows to introduce dimensionless coefficient as exponent *n* and to qualitatively compare dissolution conditions in aspects of precision and accuracy. However, it should be stressed that obtained functions differ in dimension of value *A*; therefore, physical interpretation is challenging.

It is important to note the difference between dissolution localization and electrochemical machining accuracy. Good localization provides a potential to shape with high resolution (with small unit removal) and is necessary but not sufficient to obtain high machining accuracy. Accuracy is a much wider term and is connected with the whole machining system (i.e., machine tool accuracy, clamping quality, etc.). The term localization is strictly connected with electrochemical dissolution and does not account for the above mentioned factors, therefore linking these factors with real accuracy of machining is complicated and problematic.

## 7. Research on Electrochemical Micromachining with Universal Cylindrical Electrode-Tool—Case Studies

### 7.1. Materials and Methods

This part of the paper presents research (case-studies) of electrochemical contour milling process with application of the universal cylindrical electrode-tool. This is the most adaptable—for micromachining—and the most effective kinematic variant of ECMM. Application of cylindrical electrode-tool allows to shape the part with lateral or side surface of the electrode tool. Regardless of the selected variant, the design of machining technology needs knowledge about the thickness of material removed in a single pass, efficiency, surface integrity (roughness and waviness) and dissolution localization (which defines machining accuracy). These basic factors can be determined by making simple structures such as grooves or holes. In order to machine more complicated spatial structures, it is the necessary to remove several layers of material. Therefore, a number of repetitions and directional passes of the electrode tool should be determined. The following goals of the research can be formulated: (1) to find out the basic relationship between machining parameters and technological factors (i.e., efficiency, roughness, thickness of the removed material, etc.) and (2) to check the repeatability of the process. The fulfilment of these goals allows to develop electrochemical technology for shaping 3D structures. It allows to determine the range of input parameters that could be used during further machining of spatial structures.

The research presented below was carried out on the prototype machine tool developed in Chair of Production Engineering of Cracow University of Technology. The prototype machine tool consists of the following components: mechanical unit with three axis servo drives and a control system, electrode-tool, and workpiece precision clamping based on the Swiss company EROWA solutions system, a working unit for electrolyte circulation and a power supply with process control and monitoring unit. Detailed description of the test stand is presented in [19,23].

### 7.2. Shaping of Cavity with Rectangular Cross-Section

**Figure 11:**
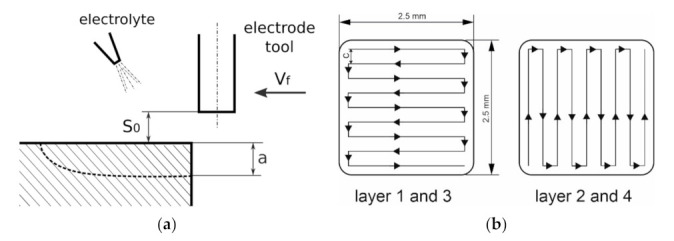
Scheme of research realization (**a**) and a trajectory of the electrode tool applied in the research (**b**). *v_f_*—tool feed, *S*_0_—size of initial interelectrode gap, *a*—thickness of the removed material, *c*—the distance between passes of the electrode tool; during machining of cavity, the electrode-tool moved on the same level.

The example reported below presents an application of electrochemical contour milling to shaping a shallow cavity. For such a micromachining process it is crucial to know the basic technological characteristics for efficiency, thickness of the removed material, and surface roughness. These parameters can be determined by machining and subsequent analysis of the grooves surface after a single electrode-tool pass. During the machining of spatial structures, in which several layers of material are removed, the distance between passes of the electrode tool, its direction and surface waviness should be determined. This allows to set the range of input parameters that could then be used during machining spatial structures. The machining parameters are summarized in Table 3 and the schemes of machining kinematics is presented in Figure 11.

**Figure 12:**
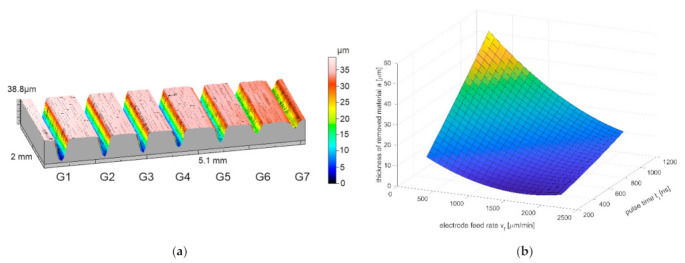
Three-dimensional image of the surface of the sample (**a**) (G1–G5: repeatable 1500 μm/min, *t_i_* = 540 ns, *U* = 20 V, *S*_0_ = 30 μm; G6: *v_f_* = 1125 μm/min, *t_i_* = 310 ns, *U* = 15 V, *S*_0_ = 20 μm; G7: *v_f_* = 1125 μm/min, *t_i_* = 310 ns, *U* = 25 V, *S*_0_ = 40 μm) and the relation between thickness of the removed material in a single electrode pass *a*, the feed rate of the electrode *v_f_* and the working pulse time *t_i_* (**b**) (the size of initial interelectrode gap *S*_0_ = 30 μm, interelectrode voltage *U* = 20 V).

In the first stage (i.e., single pass), the tests were run in accordance with principles of experimental design (DoE). It allowed to establish the optimal set of parameters for the second part of the research (i.e., cavity machining). The 3D profiles and stereometric surface images were prepared with the Taylor Hobson surface profiler (Figure 12a). It allowed to determine the depth, volume, and roughness of the obtained structures. Relations of the machining efficiency, roughness, thickness of the removed material and the volume of the removed material were investigated as a function of the pulse duration, feed rate, voltage, and thickness of the interelectrode gap. For the regression model, a second-degree polynomial was assumed as a function approximating the research object (also including the analysis of regression coefficient significance and adequacy of the polynomial). The final goal was to shape a cavity with dimensions of 2.5 mm × 2.5 mm. During the machining, the initial interelectrode gap *S*_0_ was 20 μm. The distance between passes of the electrode tool *c* was: 50 μm, 100 μm, 150 μm, and 200 μm (this gives *c*/*D* ratio equal to 0.25, 0.5, 0.75, and 1). For each value of *c*/*D* ratio, the three repetitions were made, ultimately giving 12 cavities.

**Figure 13:**
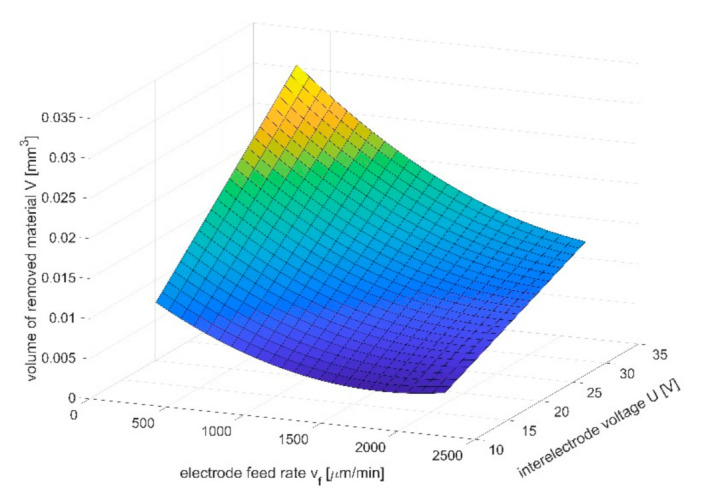
Dependence of the material volume removed in a single electrode pass on the interelectrode gap *S*_0_ and pulse time *t_i_*; feed rate *v_f_* = 1500 μm/min, interelectrode voltage *U* = 20 V.

**Figure 14:**
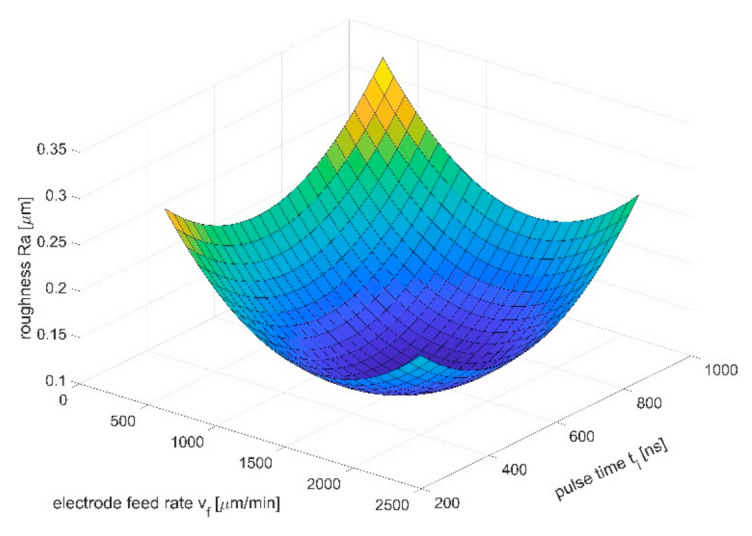
Dependence of the roughness parameter Ra on the length of the voltage pulse ti and the feed rate of the electrode *v_f_*; initial interelectrode gap *S*_0_ = 30 μm, interelectrode voltage *U* = 20 V.

The volume of the removed material as a function of feed ratio and voltage was presented in Figure 13. The volume of the removed material increases with the increase of voltage and decreases with the increase of the electrode-tool feed speed. Minima for the chosen feeds, which can be observed in the Figure 13, can be explained by poor hydrodynamic conditions in the inter-electrode gap. As it was mentioned in paragraph 3, evacuation of the dissolution products from the interelectrode gap has a very strong impact on results of the machining. In the investigated case, the highest size of the working gap can be defined as $S=a+S_{0}$. For the chosen range of parameters, when the tool-feed *v_f_* rate is >1500 μm/min, the thickness of removed material is low (*a* ≈ 10 μm), so the problem with effective electrolyte supply and flushing occurs in machining area. Solid and gaseous products of dissolution can slow down or even interrupt the process. As a result, an uneven electric current density distribution can appear and cause the surface defects or shape errors. Figure 14 shows Ra as a function of the feed rate and the pulse time. The Ra value is in range from 0.1 to 0.35 μm and its lowest value is in the center of the research plan. This is due to the fact that at low feed rates and short pulse times, dissolution lasts long enough for the roughness to increasing, as described previously. For high speeds of the electrode tool, uneven dissolution occurs, which also can lead to increased roughness. With an appropriately selected input parameters, the lowest roughness value can be obtained.

The above-mentioned technological characteristics were the basis for the selection of parameters for the second part of the research, where the influence of machining strategy (*c*/*D* ratio) on accuracy and surface quality was investigated. After the machining, the dimensions of each cavity were measured, and roughness parameters were determined. Time of the machining for each case was as follows:

- *c* = 50 μm (*c*/*D* = 0.25); time of machining 97.2 min; volumetric efficiency 0.001 mm^3^/min;
- *c* = 100 μm (*c*/*D* = 0.50); time of machining 48.6 min; volumetric efficiency 0.01 mm^3^/min;
- *c* = 150 μm (*c*/*D* = 0.75); time of machining 32.4 min; volumetric efficiency 0.015 mm^3^/min;
- *c* = 200 μm (*c*/*D* = 1); time of machining 25.2 min; volumetric efficiency 0.016 mm^3^/min.

**Figure 15:**
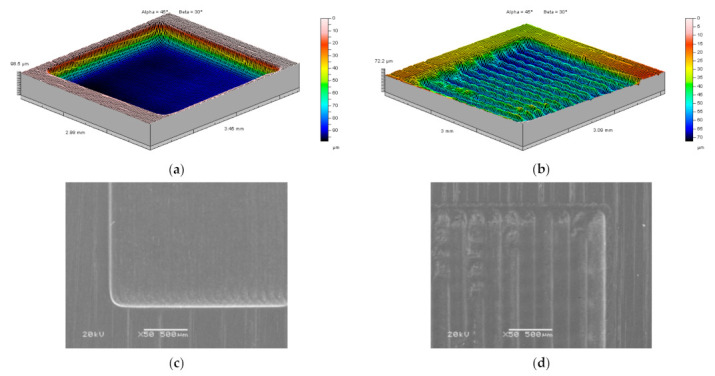
Three dimensional profiles and SEM photographs of the electrochemically milled cavities for different distances between electrode tool passes: (**a**) *c* = 50 μm (*c*/*D* = 0.25), depth 98.5 μm and (**b**) *c* = 200 μm (*c*/*D* = 1), depth 72.2 μm; (**c**) SEM photo of left bottom corner of cavity (**a**), (**d**) SEM photo of right top corner of cavity (**b**).

As an example, selected 3D profiles and SEM photographs of cavities of *c* = 50 μm (*c*/*D* = 0.25) and *c* = 200 μm (*c*/*D* = 1) were presented in Figure 15. In the case, the distance between passes is the same as the diameter of the working electrode (*c*/*D* = 1). Each pass is clearly visible as a separate row. Application of *c* equal to quarter of the tool diameter (*c*/*D* = 0.25) allows to obtain smooth bottom of the cavity. This is due to the fact that the dissolution does not occur evenly under the electrode (the electric field intensity is at its strongest in the electrode axis). This leads to a reduction of the process localization and errors of the obtained shape and uneven dissolution in the plane transverse to the feed direction.

When distance between passes of the electrode tool is equal to the diameter of the electrode (*c*/*D* = 1), there are clearly marked grooves that are traces of the passing electrode tool, and the material which was supposed to be removed is still visible at the bottom (Figure 15b). When *c* value decreases (150 μm and 100 μm), the areas of the remaining material between passages of the electrode tool are getting smaller and thus the bottom of the cavity becomes flatter. Finally, for *c*/*D* = 0.25 the shape of the cavity becomes a cuboid with a flat bottom (Figure 15a).

**Figure 16:**
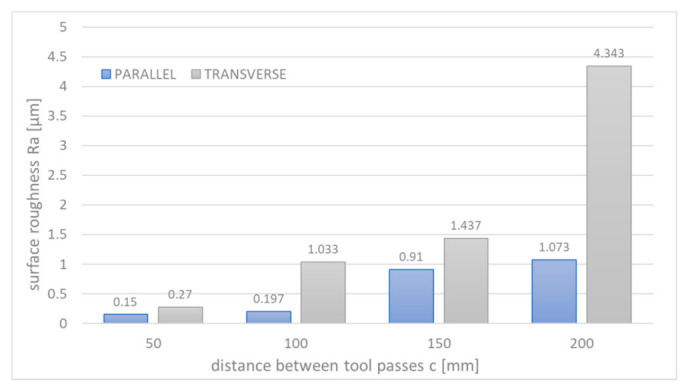
Average values of Ra changes with the distance between passes *c*; measured parallel and transverse to the electrode tool trajectory.

Based on the results presented in Figure 16, one can state that:

- the applied machining strategy and cavity profile determine surface roughness, which depends on the direction of electrode-tool feed (significant differences for Ra measured parallel and transverse to the electrode tool trajectory);
- the smaller distances between passes of the electrode tool give lower roughness of the cavity surface and flatter bottom of the cavity. In this case, the Ra = 0.15 μm (measured parallel) and Ra = 0.27 μm (measured transverse) and is in the lowest range obtained during tests for a single pass (Figure 16).

**Figure 17:**
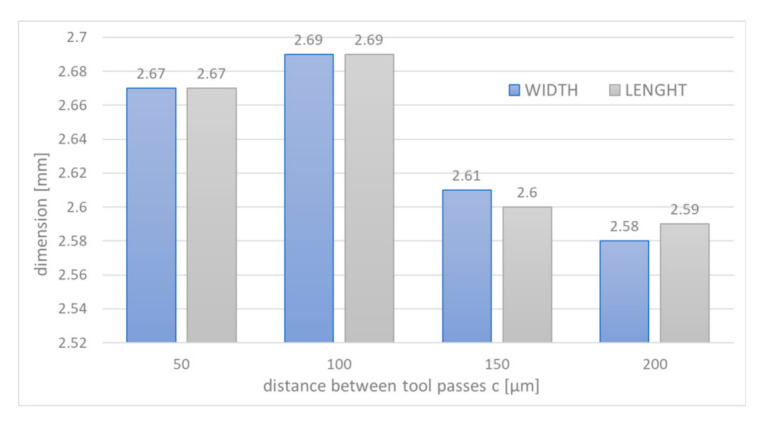
Changes of width and length of the cavity with the distance between passes of the electrode tool *c*.

**Figure 18:**
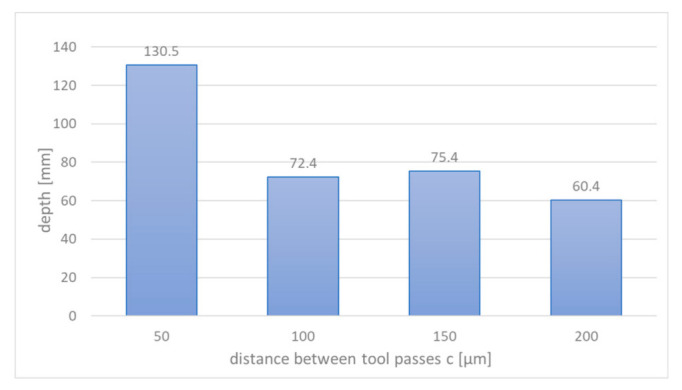
Changes of depth of the cavity with the distance between passes of the electrode tool *c*.

In the Figure 17, the average length and width of obtained cavities were presented. As one can see, width and length differ slightly (less than 10%) from the nominal value (2.5 mm). This indicates the need for electrode-tool trajectory correction. The obtained cavity depth varies from 60 to 130 μm (Figure 18). The process parameters (voltage level, voltage pulse time) have impact on the obtained results. However, it is worth to note that during the machining of the cavity, the electrode-tool was moved on the same level, so the differences in depth are mainly related to distances between tool passes. A consequence of using cylindrical electrode tool is an uneven dissolution in cross section perpendicular to the feed direction (the thickness of the removed material decreases with the distance from the tool axis). Increasing the distance between passes of the electrodes causes the areas of the unremoved material to appear, which is due to the reduction of dissolution area to the area of the forehead of the electrode only.

### 7.3. Example of Application of Rotating Cylindrical Electrode Tool

**Figure 19:**
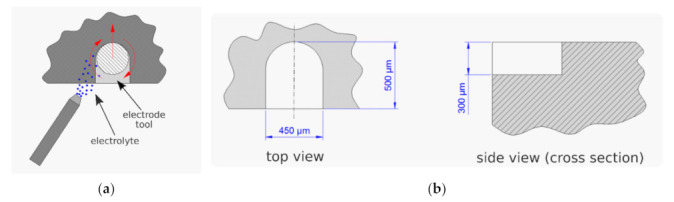
Scheme of the rotary ECM process with: (**a**) co-rotating electrolyte supply; (**b**) top and side view of the cavity with nominal dimensions [33].

**Figure 20:**
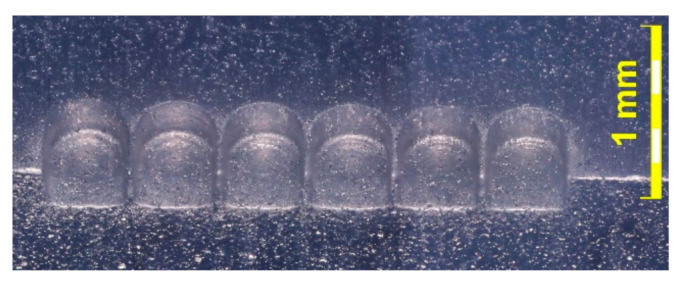
Cavities machined for tests of repeatability [33].

The second example of electrochemical micromachining technology development also focuses on application of cylindrical electrode tool, however in this case shaping was executed with a sidewall surface of rotating electrode tool (rotary ECM, Figure 19a). This research was also conducted in Chair of Production Engineering of Cracow University of Technology [33] and the most important results of this work were presented below. In this ECMM variant, the electrolyte can be supplied counter-rotating or co-rotating with the rotation of the electrode. The preliminary studies showed that counter-rotating supply is ineffective, and the machining is accompanied by frequent discharges (their number increases with depth). Therefore, further tests were conducted for co-rotating electrolyte supply and with the machining parameters presented in Table 4. Application of a very low pulse duty factor, i.e., $\frac{t_{i}}{t_{i}+t_{p}}=$ 0.091, and low concentration electrolyte, i.e., 1% water solution of NaNO_3,_ allows to conduct the reliable machining with a very thin interelectrode gap. However, it necessitates the need to use the tool feed rate lower than 100 μm/min. Therefore, the efficiency of the process is very low. Considering the constant electrical parameters, the dimensions of the obtained cavity depends mainly on feed rate, which affects electric current density and the machining gap size. Therefore, in order to obtain the cavity with the dimensions: width 450 μm, length 500 μm, and depth 300 μm, as presented in Figure 19b, the tests for various feed values were prepared. The machining was stable for electric current in the range from 400 to 700 mA. Above this threshold a short circuit occurred and, for the electric current equal 1 A, the short circuit was permanent. Finally, for feed rate 39 μm/min, the following dimensions were obtained: width 496.8 μm, length 500.8 μm, and depth 291.7 μm and this feed value was selected for further tests. It was assumed that relatively high width error results from electrode tool eccentricity of clamping. Therefore, the clamping was corrected, and the test was repeated six times to check shaping repeatability. Table 5 and Figure 20 show the result of the machining for six subsequent repetitions. The widths of the cavities obtained differ by less than 1%, which proves very good repeatability of the process.

**Figure 21:**
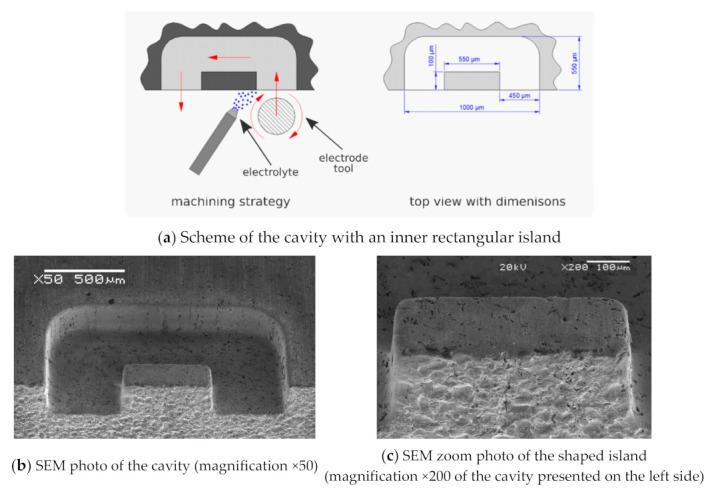
Schemes (**a**) and photographs (**b**,**c**) of the machined cavity with an inner rectangular island [33].

The next step was to apply the above presented results to set up the machining of a cavity with an inner rectangular island (see Figure 21) in rotary ECMM kinematics. The following dimensions were assumed for the island: length—550 μm and width—100 μm. However, the final obtained shape was different from the assumed one. The length of the machined island was 547 μm and was different from the assumed one by less than 1%, but the width was 167.8 μm which was 50% greater than the assumed one. This difference can be explained by the difficulty of supplying the electrolyte to the interelectrode gap and the problem of the evacuation of electrochemical dissolution products from the interelectrode gap and the cavity. It should be noted that the position of electrolyte nozzle was fixed, so the change of the feed direction changed the electrolyte flow conditions in the gap.

## 8. Summary

Electrochemical machining offers great opportunities for the production of micro and macro components. This is due to the fact that the efficiency does not depend on the mechanical properties of the machined material. In ECMM, the volume accumulation of hydrogen is not significant; therefore, the probability that the process itself would introduce any structural changes in the surface layer is negligible. On the other hand, the problems with localization of the process, and removal of the erosion products from the interelectrode gap, are the limitations of this process. The use of pulse power supplies and a proper selection of parameters allow to obtain satisfactory effects of machining using this method.

The physicochemical and technological aspects supported by the authors’ research on the development of electrochemical machining of 3D microparts were presented in this paper. The specificity and characteristics of electrochemical micromachining and the trends of the current research were presented. Conditions of electrochemical dissolution were discussed and the possibilities of improvement of the shaping accuracy were indicated. The discussion of ECM process adaptation to micromachining focused on problems with dissolution localization. Two approaches to measurement of the machining localization factor were presented. Theoretical analysis in this area was supported by an empirical experiment. The objective of this experiment was to find the characteristic *q*(*S*,*U*) which describes charge density as a function of the gap size *S* and the interelectrode voltage U. This relationship allows to link technological parameters with dissolution localization. Nevertheless, it is important to highlight that a more precise association of these factors with accuracy of machining warrants further research. The goal of the discussion was to elucidate problems of ECMM technology design and development and to describe the processes and variants of electrochemical micromachining. The application of voltage pulses (Pulse Electrochemical Machining, PECM) holds most promise in the area of precise manufacturing methods. This kind of machining can be conducted with activation limits (pulse time *t_i_* < 500 ns) and with mass transport limitations (pulse time 1 μs < *t_i_* < 100 μs). The two PECM variations were characterized by two different dissolution localization principles, different areas of application and different scaling possibilities.

Finally, the case studies covered application of pulse electrochemical micromachining (PECMM) in electrochemical contour milling (shaping shallow cavity) and shaping conducted with sidewall surface of rotating tool (rotary PECMM). In the first case, the primary test with machining of simple shapes (grooves) allowed to select the most effective process parameters for further process development. These enabled to determine the efficiency, surface roughness of the obtained parts, and the localization of the process. Shape and size accuracy depend also on the appropriate machining strategy related to the choice of the tool trajectory and the method of electrolyte feeding to the machining area. This was presented on the example of roughness the value of which depends not only on the set of machining parameters, but also on the selection of the distance between passes. This value is resultant of the process parameters and the method of its implementation. As a conclusion, the obtained results show that microelectrochemical machining with its precision and accuracy is suitable for industrial application.

The study shows the fundamentals of electrochemical machining technology development with special attention to micromachining applications. Basic parameters such as pulse time, voltage, tool feed rate influence the efficiency, and localization and roughness, but the machining strategy is very important. In case of electrochemical shape machining using cylindrical electrode tool, it is very important to choose the right strategy of electrode tool trajectory design as well as the standard parameters such as pulse time, feed rate, inter electrode voltage, and inter electrode gap size that directly affect efficiency and localization. During the machining using advancing (milling kinematics), it is crucial to select the proper distance between passes, as well as the direction of removing of next layer in relation to the previous one. Application of short voltage pulses improves localization of the dissolution. It is also important that when the distance between passes of the electrode-tool is comparable to the diameter of the electrode, the result is low surface quality and geometrical accuracy. Obtaining shape of high accuracy requires application of a small distance between passes of the electrode tool and, therefore, an extended machining time. A final surface pass has a huge impact on the final shape of the manufactured part. Consequently, it is critical to the choose a suitable strategy to optimize the entire process warranting acceptable product geometry and manufacturing time. The whole process can be divided into two steps: rough machining where most of the material is removed in short time but accuracy is low, and finishing machining where the material is removed in selected areas and the accuracy is very high. Another solution could be choosing the proper electrode tool trajectory so that the resulting shape would fit expected geometry without additional operations. This technique of machining can be applied for production of moulds, dies and tools used in micromachining processes.

Another essential aspect of the manufacturing process is its repeatability. The results of the research using rotary PECMM example showed that despite the problems with effective electrolyte supply and gap flushing, the satisfactory results can be obtained. Preliminary research made it possible to determine the optimal process parameters to be used in further machining. Dimensions of the machined cavities differed from those assumed by less than 1%, which can be considered a good level of repeatability of the process. Presented procedures can be effectively used in the production of microforming tools, prototypes, and short series of microparts.

## Figures and Tables

**Table 1:** Summary of calculations performed on the basis of the equation *q*(*S*,*U* = *const*) for 5% water solution of NaNO_3_, *S*_1_ = 0.01 mm, *S*_2_ = 0.1 mm.

*U* (V)	*q*_1_ (μC/mm^2^)	*q*_2_ (μC/mm^2^)	*v*_*n*1_ (mm/s)	*v*_*n*2_ (mm/s)	*dv/dS* (1/s)	*v*_*n*1_/*v*_*n*2_	*n*
6	0.785	0.458	0.0021	0.0012	0.0099	1.71	0.234
9	1.361	0.758	0.0037	0.0021	0.0183	1.80	0.254
12	2.175	1.156	0.0059	0.0032	0.0309	1.88	0.275
15	3.206	1.625	0.0087	0.0044	0.0479	1.97	0.295
18	4.365	2.105	0.0119	0.0057	0.0685	2.07	0.317
25	6.494	2.810	0.0177	0.0077	0.1126	2.31	0.364

**Table 2:** Summary of calculations performed on the basis of the equation *q*(*S*,*U = const*) for 5% water solution of NaNO_3_, *S*_1_ = 0.01 mm, *S*_2_ = 0.02 mm.

*U* (V)	*q*_1_ (μC/mm^2^)	*q*_2_ (μC/mm^2^)	*v*_*n*1_ (mm/s)	*v*_*n*2_ (mm/s)	*dv/dS* (1/s)	*v*_*n*1_/*v*_*n*2_	*n*
6	0.785	0.719	0.0021	0.0020	0.0179	1.09	0.126
9	1.361	1.240	0.0037	0.0034	0.0330	1.10	0.134
12	2.175	1.972	0.0059	0.0054	0.0554	1.10	0.141
15	3.206	2.892	0.0087	0.0079	0.0856	1.11	0.149
18	4.365	3.909	0.0119	0.0107	0.1244	1.12	0.159
25	6.494	5.755	0.0177	0.0157	0.2015	1.13	0.174

**Table 3:** Input and constant machining parameters used during test on shaping of cavity with rectangular cross-section.

Machining Parameter	Preliminary Research (Single Pass)	Cavity Machining
Pulse time on *t_i_* [μs]	0.08–1	1
Pulse time off *t_p_* [μs]	1	10
Feed rate *V_f_* [μm/min]	375–2250	1500
Initial interelectrode gap thickness, *S*_0_ [μm]	10–50	20
Interelectrode voltage *U* [V]	10–25	20
Electrolyte	1.5% NaNO_3_ aqueous solution	
Temperature of electrolyte *T* [°C]	23	
Workpiece material	1.4301 stainless steel	
Material of electrode tool	tungsten	
Diameter of electrode *D* [μm]	200	
Dimensions of cavity [μm × μm]	-	2700 × 2700
The distance between passes of the electrode tool *c* [μm]	-	50, 100, 150, 200
Number of removed layers	1	4

**Table 4:** Input and constant machining parameters for rotary ECMM tests [33].

Material of Electrode Tool	Copper
Workpiece material	1.4301 stainless steel
Diameter of electrode *D* [μm]	400
Initial interelectrode gap thickness *S*_0_ [μm]	100
Electrolyte	1% water solution of NaNO_3_
Tool rotation *TR* [1/min]	500
Feed rate *V_f_* [μm/min]	39 (tested: 50, 55, 60, 65, 80)
Interelectrode voltage *U* [V]	20
Pulse time on *t_i_* [μs]	1
Pulse time off *t_p_* [μs]	10

**Table 5:** Dimensions of machined cavities obtained in tests of repeatability [33].

Number of Cavity	Width (μm)	Mean Width (μm)	Standard Deviation of Width (μm)	Range (μm)
N1	449.1	449.1	4.8	12.5
N2	446.0			
N3	445.9			
N4	449.1			
N5	446.0			
N6	458.4			

## Data Availability

The data presented in this study are available on request from the corresponding author.

## Abbreviations

*a*	thickness of removal material
*c*	the distance between the passes of the electrode tool
*D*	diameter
*j*	electrical current density
*i*	electrical current intensity
*n*	localization factor
*Q*	electrical charge
*q*	charge density
*S*	interelectrode gap thickness
*S*_0_	initial interelectrode gap thickness
*S_L_*	limiting interelectrode gap thickness
*T*	temperature
*t*	time
*t_i_*	voltage pulse time
*t_p_*	pause time between voltage pulses
*U*	interelectrode voltage (electric potential)
*V_p_*	volume of material removed during one pulse
*v_n_*	dissolution speed (ratio)
*v_f_*	electrode tool feed rate
*ηk_v_*	electrochemical machinability
*κ*	the electrolyte electrical conductivity
ECM	Electrochemical Machining
ECMM	Electrochemical Micro Machining
PECM	Pulse Electrochemical Machining
PECMM	Pulse Electrochemical Micro Machining
UR	Unit Removal
